# Complete Genome Sequences of Four Campylobacter jejuni Strains Isolated from Retail Chicken Meat and Broiler Feces

**DOI:** 10.1128/mra.00898-22

**Published:** 2022-09-15

**Authors:** Sabin Poudel, Tianmin Li, Mark A. Arick, Chuan-Yu Hsu, Adam Thrash, Anuraj T. Sukumaran, Pratima Adhikari, Aaron S. Kiess, Li Zhang

**Affiliations:** a Department of Poultry Science, Mississippi State University, Mississippi State, Mississippi, USA; b Department of Biomedical Sciences, City University of Hong Kong, Kowloon, Hong Kong, China; c Institute for Genomics, Biocomputing, and Biotechnology, Mississippi State University, Mississippi State, Mississippi, USA; d Prestage Department of Poultry Science, North Carolina State University, Raleigh, North Carolina, USA; University of Rochester School of Medicine and Dentistry

## Abstract

Campylobacter jejuni is the leading pathogen that causes foodborne infections. Here, we report the complete genome sequences of four C. jejuni strains isolated from retail chicken meat and broiler feces samples. Genes encoding type VI secretion and antibiotic resistance were detected among these isolates.

## ANNOUNCEMENT

Campylobacteriosis, caused by Campylobacter jejuni, is one of the major foodborne infections that cause human gastroenteritis, and poultry is considered the major reservoir host (1–6). Therefore, to understand the variation in molecular characteristics of C. jejuni poultry isolates, whole-genome sequencing was performed to determine detailed information about the virulence and antimicrobial resistance genes (6–8).

Four C. jejuni strains isolated and identified in our previous study (9) were further utilized. Each C. jejuni strain was cultured in Bolton broth (Thermo Fisher Scientific, USA) and incubated under microaerophilic conditions for 48 h at 42°C; genomic DNA was then extracted using the GeneJET genomic DNA purification kit (Thermo Fisher Scientific). For long-read sequencing, genomic DNA was fragmented using a g-TUBE device (Covaris, USA) and the fragments size selected to a mean of 8 to 12 kb using AMPure XP beads (Beckman Coulter, USA) for library preparation. A multiplexing library pool was prepared and barcoded using a genomic DNA ligation kit (SQK-LSK109) and the native barcoding genomic DNA kit (SQK-NBD104), respectively; the library was sequenced on an R9.4 MinION flow cell using the Nanopore GridION sequencer (Oxford Nanopore Technologies, Oxford, UK) for 48 h. For short-read sequencing, Illumina TruSeq DNA PCR-free (insert size, 350 bp) library preparation and sequencing using the paired-end 150-bp (PE150) method was performed by Novogene on the HiSeq X Ten sequencer (Illumina, USA). The raw Nanopore reads were assembled into contigs using Canu v1.9 (10). The first 1,000 bp of each contig was mapped to the entire contig using blastn from the BLAST+ v2.9.0 tool suite (11); then, the overlapping regions were removed using SAMtools v1.9 (12). The start of each circular contig that had an overlapping region was moved upstream of the *dnaA* gene, when present, or upstream of the gene closest to the middle of the contig using Circlator v1.5.5 (13). The junction site of circular contigs was verified using PCR amplification and Sanger sequencing (14). The Illumina and Nanopore reads were mapped to the assembled contigs using BWA v0.7.17-r1188 (15) and minimap2 v2.17 (16), respectively. Any contig without supporting Illumina data was removed. The remaining contigs were polished using Pilon v1.23 (17). The genome sequences were annotated using Prokka v1.13 (18).

The genome sizes and number of protein-coding DNA sequences of the isolates range from 1.63 Mb to 1.84 Mb and 1,421 to 1,552, respectively, and two isolates (MS2005 and MS2058) contain one plasmid each. The respective fold coverages for Illumina and Nanopore ranged from 541 to 864 and 440 to 851, and the *N*_50_ values ranged from 9,302 to 10,256 bp, respectively. *In silico* prediction of antimicrobial resistance genes using CARD v3.1.0 (19) identified *aph(3′)-IIIa*, *sat-4*, and *bla*_OXA-61_ genes in the chromosomes, whereas plasmid pMS2058 contains the *tet*(O) gene. Using SecReT6 (20), the presence of type VI secretion genes was predicted in the chromosomes of three C. jejuni strains MS2005, MS2058, and MS2167 (Table 1). The genome information obtained in this study can be further utilized for the identification of potential vaccine candidates and functional analysis of type VI secretion genes.

**TABLE 1:** Summary of genome assembly and genotype characteristics of four C. jejuni isolates

Strain	Source	GenBank accession no.	Size (bp)	Illumina		Nanopore			GC content (%)	No. of CDSs^*a*^	ARG(s)^*b*^	Virulence genes
				No. of reads	Avg per-base coverage (×)	No. of reads	Avg per-base coverage (×)	*N*_50_ (bp)				
C. jejuni MS2005^*c*^	Retail chicken meat	CP084080	1,757,242	3,476,551	571	164,000	736	10,111	30.5	1,541	*aph(3′)-IIIa*, *sat-4*, *cmeR*	*tssA*, *tssB*, *tssC*, *tssD*, *tssE*, *tssF*, *tssG*, *tssI*, *tssJ*, *tssK*, *tssL*, *tssM*
C. jejuni MS2005^*d*^	Retail chicken meat	CP084081	5,208		6,746		405		28.5	6		
C. jejuni MS2058^*c*^	Retail chicken meat	CP084082	1,758,823	5,455,985	890	104,000	472	10,256	30.5	1,552	*aph(3′)-IIIa*, *sat-4*, *cmeR*	*tssA*, *tssB*, *tssC*, *tssD*, *tssE*, *tssF*, *tssG, tssJ*, *tssK*, *tssL*, *tssM*
C. jejuni MS2058^*d*^	Retail chicken meat	CP084083	40,825		1,086		231		28.5	41	*tet*(O)	
C. jejuni MS2074^*c*^	Retail chicken meat	CP084084	1,629,343	5,376,179	986	144,000	691	9,909	30.7	1,491	*bla*_OXA-61_, *cmeR*	
C. jejuni MS2167^*c*^	Broiler feces	CP084085	1,711,301	5,762,419	1,007	220,000	951	9,302	30.5	1,491	*cmeR*	*tssA*, *tssB*, *tssC*, *tssD*, *tssE*, *tssF*, *tssG*, *tssI*, *tssJ*, *tssK*, *tssL*, *tssM*

a CDSs, protein-coding DNA sequences.

b ARG(s), antimicrobial resistance gene(s).

c Chromosome.

d Plasmid.

### Data availability.

The genome sequences and raw data are available at NCBI GenBank under the BioProject accession number PRJNA655459. The specific parameters and code used for sequencing can be found at https://github.com/IGBB/campylobacter_jejuni.
